# Epidemiological and geodemographic patterns of scorpionism in Ecuador: A nationwide analysis (2021–2024)

**DOI:** 10.1016/j.toxcx.2025.100218

**Published:** 2025-03-08

**Authors:** Jorge Vasconez-Gonzalez, Juan S. Izquierdo-Condoy, Camila Miño, María de Lourdes Noboa-Lasso, Esteban Ortiz-Prado

**Affiliations:** a One Health Research Group, Faculty of Health Science, Universidad de Las Americas, Quito, Ecuador; b Program in Occupational Safety and Health, The University of Porto, Porto, Portugal

**Keywords:** Scorpion sting, Epidemiology, Scorpion envenomation, Public health, Ecuador

## Abstract

**Background:**

Approximately 1.2 million scorpion stings are reported globally each year, resulting in an estimated 3000 deaths. Of the 2500 known scorpion species, about 40 are considered medically significant. In Ecuador, where at least 47 scorpion species exist, information on scorpion stings remains scarce.

**Methods:**

A nationwide cross-sectional analysis was conducted on all officially reported cases of scorpion stings documented in the epidemiological surveillance reports from the Ministry of Public Health in Ecuador between 2021 and 2024.

**Results:**

A total of 1633 cases were identified, with women accounting for 52% of cases (n = 849). The highest incidence was observed among children aged one to four years old, with rates of 18.16 and 19.11 per 100,000 inhabitants for males and females, respectively. Geographically, the Amazon region was the most affected, with the province of Morona Santiago reporting the highest incidence at 284.14 cases per 100,000 inhabitants.

**Conclusion:**

Scorpion stings represent a significant and underreported public health threat in Ecuador. This study highlights the considerable disease burden, particularly in specific regions of the country, and underscores the urgent need for targeted public health interventions and policy changes, including the local production of antivenoms.

## Highlights

• Around 1.2 million scorpion stings are reported annually worldwide.
• In Ecuador (2021-2024), 1,633 scorpion stings were reported, mostly in women.
• The Amazon region has the highest incidence of scorpion stings.
• Scorpions exist in all Ecuadorian provinces, but no cases were recorded in the Galápagos Islands in the past 4 years.

## Graphical abstract

**Figure:**
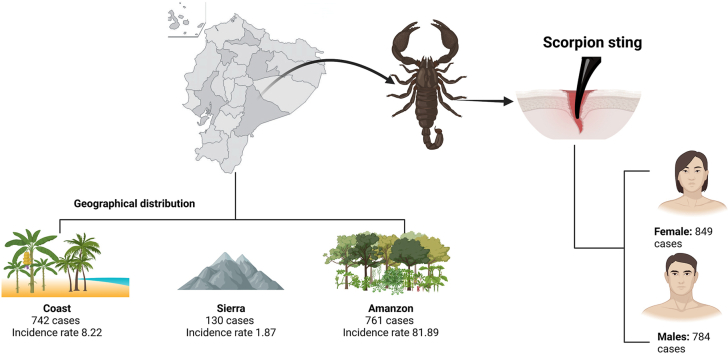

## 1 Introduction

Scorpionism, or envenoming by medically significant scorpions, is a significant public health concern worldwide, yet it receives little attention despite the wide distribution of scorpions across the planet (Chippaux and Goyffon, 2008). While the medical consequences of scorpion stings do not always require treatment, they pose a considerable health risk, particularly in tropical and subtropical regions where their distribution is prevalent (Santos et al., 2016). This risk is especially pronounced for vulnerable populations, such as young children. Of the approximately 2500 scorpion species identified globally, around 40 are recognized as hazardous to humans due to their potent venom (Du et al., 2024). Annually, scorpion stings affect over 1.2 million individuals, with children experiencing more severe and frequently fatal outcomes, particularly in underdeveloped regions with delayed access to emergency medical care (Chippaux and Goyffon, 2008). Scorpion envenomation can range from mild to severe and is responsible for at least 3000 deaths annually worldwide, making it the second leading cause of fatalities caused by venomous animals, surpassed only by snakebites (Chippaux, 2015).

Scorpion stings typically occur during outdoor activities, as scorpions hide in tree bark, under rocks and logs, or inside burrows (Lacerda et al., 2022). They also invade indoor spaces, seeking cooler temperatures in shoes, bags, or bed linens (Alhelail et al., 2024). Most scorpion envenomation cases occur due to unintentional contact with the animal, prompting a defensive reaction that results in venom injection. The venomous apparatus of scorpions is located in the telson, the final segment of the post-abdomen, and comprises a venom vesicle containing paired glands (Furtado et al., 2020; Santos et al., 2016). The venom within the human body, primarily composed of neurotoxic peptides, rapidly disseminates into the extravascular compartment within 2 h, triggering sympathetically and parasympathetically mediated symptoms depending on the scorpion species and venom dose (Chippaux and Goyffon, 2008; Furtado et al., 2020).

Clinical manifestations of scorpion envenomation vary widely and are classified using Abroug's classification system into three severity levels: Level I (localized symptoms), Level II (systemic symptoms), and Level III (life-threatening symptoms) (Chippaux and Goyffon, 2008; Jarrar and Al, 2008; Santos et al., 2016). The severity and progression of symptoms depend on multiple factors, including the scorpion species, number of stings, and the patient's vulnerability, particularly in pregnant women, children, the elderly, and those with pre-existing health conditions (Chippaux, 2012). Treatment typically involves symptomatic management and the administration of antivenom, although mandatory case reporting could enhance clinical outcomes and strengthen public health responses (Chippaux, 2015; Santos et al., 2016).

In South America, the *Tityus* genus represents the primary scorpion threat to humans (Santos et al., 2016). Species such as *Tityus stigmurus*, *Tityus serrulatus*, *Tityus obscurus*, and *Tityus bahiensis* have proliferated in urban areas, contributing to a substantial increase in scorpion envenomation cases in Brazil (Guerra-Duarte et al., 2023a). By 2017, Brazil recorded over 120,000 annual cases, representing a more than threefold increase in a decade, surpassing case numbers for many neglected tropical diseases (Guerra-Duarte et al., 2023b).

In Ecuador, despite the presence of medically significant scorpion species, epidemiological data on scorpionism remain scarce. The absence of comprehensive studies hinders the development of evidence-based clinical and public health interventions. This study aims to address the gap in epidemiological knowledge by analyzing the epidemiological profile and spatial distribution of scorpion envenomation in Ecuador.

## 2 Methodology

### 2.1 Study design

This cross-sectional, countrywide study aimed to analyze the demographic and spatial distribution patterns of scorpion stings in Ecuador during the period 2021 to 2024.

### 2.2 Population and setting

The study was conducted in Ecuador, a country geographically divided into four distinct regions: the Coast, the Sierra, the Amazon basin and the Galapagos Islands. Politically, Ecuador comprises 24 provinces and spans an area of 283,560 km^2^ (Instituto Nacional de Estadística y Censos INEC, 2013). The country is characterized by diverse climates: the Coast experiences average temperatures of 24–25 °C, the Sierra 8–20 °C, the Amazon 24–25 °C, and the Galapagos Islands 23 °C (Varela and Ron, 2022). According to the National Institute of Statistics and Censuses (INEC), Ecuador had a total population of 17,938,986 inhabitants in 2024.

### 2.3 Data source

Data on scorpion stings were retrieved from the Epidemiological Gazettes published by the Ministry of Public Health (MSP) of Ecuador. These reports consolidate information collected through the Integrated Epidemiological Surveillance System (SIVE-Aleta) (MPS, 2024). The data, which are anonymized and publicly available, can be accessed at: https://www.salud.gob.ec/gaceta-efectos-toxicos/

### 2.4 Data analysis

The analysis included variables such as age, sex, place of residence, and year of medical care. Incidence rates were calculated per 100,000 inhabitants and standardized by sex, age, and geographic location using population projections from INEC. Cases were classified into eight distinct age groups to assess demographic patterns comprehensively.

### 2.5 Ethical considerations

This study received approval from the Human Research Ethics Committee of the Universidad de Las Américas (CEISH-UDLA) under the research protocol titled *“Global Impact of Various Epidemiological Diseases in Ecuador: A Socio-Demographic Analysis of National Morbidity and Mortality”* (protocol code 2023-EXC-008). The committee determined that the project complies with current legal and ethical standards.

## 3 Results

### 3.1 Overall cases and mortality

**Fig. 1:**
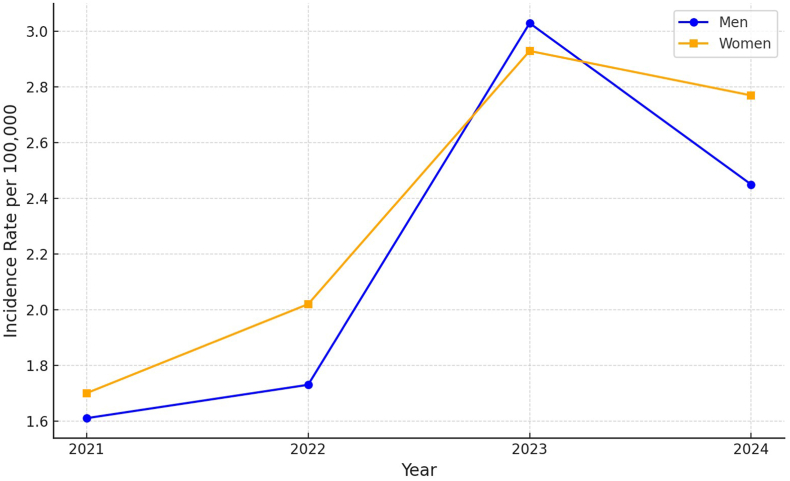
Scorpion sting incidence rates in Ecuador by Gender (2021–2024).

From 2021 to 2024, a total of 1633 scorpion sting cases and 5 deaths were reported nationwide. Males accounted for 48% (n = 784) of cases, with an incidence rate of 8.83 per 100,000 inhabitants, while females accounted for 52% (n = 849), with a higher incidence rate of 9.42 per 100,000 inhabitants. The temporal trends in scorpion sting incidence were similar between sexes, with a notable increase in 2023 when the incidence rates were 3.03 per 100,000 for males and 2.93 per 100,000 for females (Fig. 1).

### 3.2 Incidence and mortality

Females consistently experienced a higher incidence rate (9.42 per 100,000) compared to males (8.83 per 100,000). The highest incidence rates in both sexes were observed in the 1–4-year-old age group, with 18.16 and 19.11 cases per 100,000 inhabitants for males and females, respectively. Regarding mortality, 1 death was recorded in males and 4 in females. The mortality rates were 0.011 per 100,000 for males and 0.044 per 100,000 for females (Table 1).

**Table 1:** Incidence rates of scorpion stings per 100,000 inhabitants by age and sex in Ecuador (2021–2024).

Age Group	Women			Men		
(years)	Cases (n)	%	Incidence	Cases (n)	%	Incidence
<1	2	0.24%	1.51	16	2.04%	11.60
1–4	103	12.13%	19.11	105	13.39%	18.66
5–9	93	10.95%	12.94	108	13.78%	14.39
10–14	72	8.48%	9.35	92	11.73%	11.41
15–19	81	9.54%	10.20	53	6.76%	6.39
20–49	349	41.11%	8.63	300	38.27%	7.43
50–64	93	10.95%	7.76	78	9.95%	7.12
>64	56	6.60%	6.86	32	4.08%	4.84
Total	849	52,0	9.42	784	48,0%	8.83

### 3.3 Geographic distribution

**Fig. 2:**
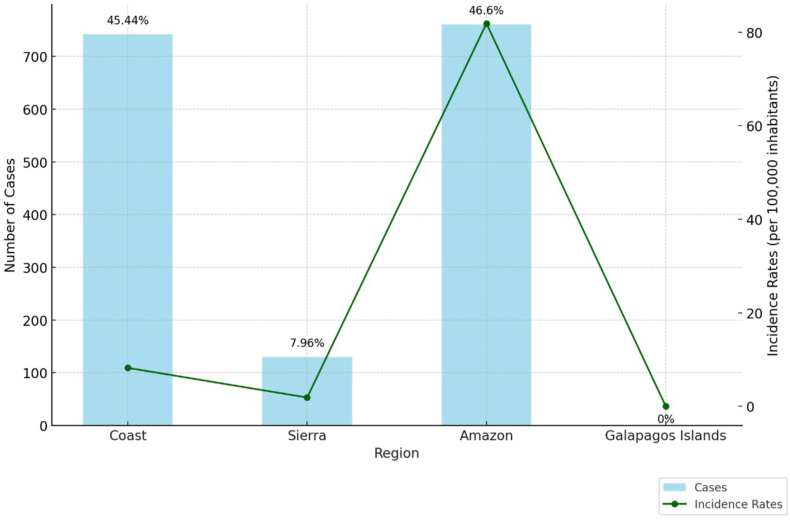
Scorpion sting cases and incidence rates per 100,000 inhabitants by region in Ecuador (2021–2024).

The Amazon region accounted for the majority of cases (46.60%, n = 761), followed by the Coast region (45.44%, n = 742). No cases were reported from the Galapagos Islands. The highest regional incidence was recorded in the Amazon (81.89 per 100,000), followed by the Coast (8.22 per 100,000) (Fig. 2).

**Fig. 3:**
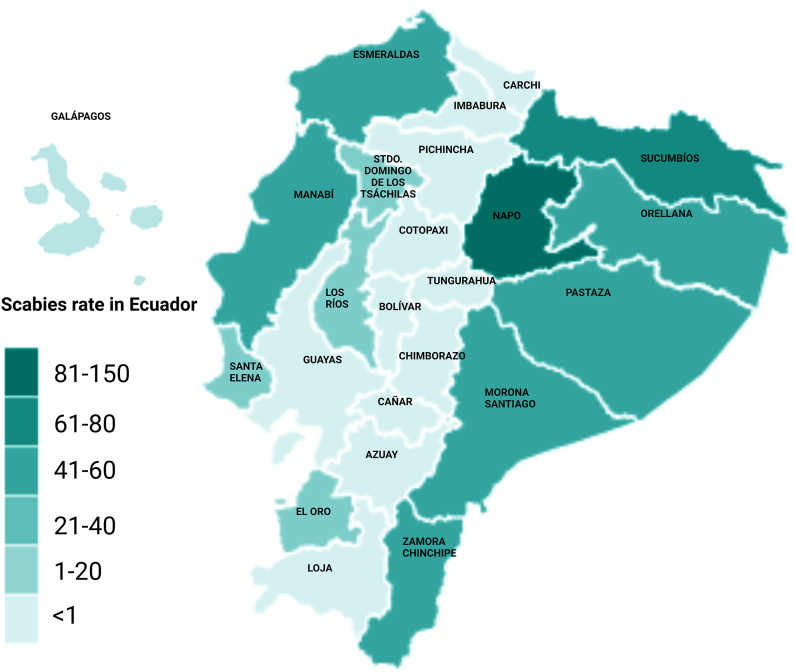
Scorpion sting incidence rates per 100,000 inhabitants by province in Ecuador (2021–2024).

At the provincial level, Morona Santiago had the highest incidence rate (284.14 per 100,000), followed by Sucumbíos (55.77 per 100,000) and Pastaza (29.49 per 100,000), all located in the Amazon region. The lowest incidence rates were observed in Azuay (0.50 per 100,000), Cotopaxi (0.45 per 100,000) and Tungurahua (0.35 per 100,000). No cases were reported from Galápagos, Carchi, or Cañar provinces (Fig. 3).

## 4 Discussion

Scorpionism is a significant public health issue in Ecuador, a country located in a region known for the greatest alpha diversity of scorpions globally, which includes northeastern Peru, southern Colombia, and the Upper Amazon region of Brazil (Ochoa-Andrade et al., 2022; Román et al., 2018). Ecuador is home to 47 scorpion species across eight genera and five families (Table 2), distributed among its four geographic regions: Coast, Sierra, Amazon, and Galapagos Islands (Brito and Borges, 2015).

**Table 2:** Scorpion species and their geographical distribution in Ecuador. Adapted from (Brito and Borges, 2015).

Region	Family	Genus	Species
Coast	*Bothriuridae*	*Brachistosternus*	*Brachistosternus ehrenbergii*
	*Buthidae*	*Ananteris*	*Ananteris festae*
			*Ananteris mariaelenae*
		*Centruroides*	*Centruroides gracilis*
			*Centruroides margaritatus*
		*Tityus*	*Titus asthenes*
			*Tityus ecuadorensis*
			*Tityus julianae*
			*Tityus pugilator*
			*Tityus timendus*
	*Caraboctonidae*	*Hadruroides*	*Hadruroides doriai*
			*Hadruroides elenae*
			*Hadruroides maculatus*
	*Chactidae*	*Teuthraustes*	*Teuthraustes rosenbergi*
			*Teuthraustes wittii*
Sierra	*Bothriuridae*	*Brachistosternus*	*Brachistosternus pegnai*
	*Buthidae*	*Ananteris*	*Ananteris festae*
		*Centruroides*	*Centruroides margaritatus*
		*Tityus*	*Tityus crassicauda*
			*Tityus ecuadorensis*
			*Tityus forcípula*
			*Tityus intermedius*
			*Tityus pugilator*
			*Tityus roigi*
	*Caraboctonidae*	*Hadruroides*	*Hadruroides moreti*
			*Hadruroides udvardyi*
	*Chactidae*	*Chactas*	*Chactas mahnerti*
		*Teuthraustes*	*Teuthraustes atramentarius*
			*Teuthraustes camposi*
			*Teuthraustes gervaisii*
			*Teuthraustes lojanus*
			*Teuthraustes oculatus*
			*Teuthraustes ohausi*
			*Teuthraustes rosenbergi*
			*Teuthraustes simonsi*
			*Teuthraustes whymperi*
			*Teuthraustes wittii*
Amazon	*Buthidae*	*Ananteris*	*Ananteris ashmolei*
		*Tityus*	*Tityus asthenes*
			*Tityus bastosi*
			*Tityus demangei*
			*Tityus gasci*
			*Tityus jussarae*
			*Tityus silvestris*
			*Tityus ythieri*
	*Chactidae*	*Chactas*	*Chactas mahnerti*
			*Chactas moreti*
			*Chactas yaupi*
		*Teuthraustes*	*Teuthraustes dubius*
			*Teuthraustes festae*
	*Troglotayosicidae*	*Troglotayosicus*	*Troglotayosicus vachoni*
Galapagos Islands	*Buthidae*	*Centruroides*	*Centruroides exsul*
	*Caraboctonidae*	*Hadruroides*	*Hadruroides galapagoensis*

Research on scorpion stings in Ecuador is notably scarce. This study represents one of the first nationwide epidemiological analyses of scorpion stings. A review of the available literature identified only five published articles on the subject in Ecuador, as summarized in Table 3. Of these, the majority (n = 3) are case reports, underscoring the limited exploration and study of this critical public health issue within the country.

**Table 3:** Summary of scientific articles on scorpion stings in Ecuador.

Author	Year	Type of study	Participants	Main Outcomes
Borges et al. (Borges et al., 2015)	2015	Case report	5	Five intra-domiciliary cases of scorpion envenomation in victims aged 1.9–16 years old, including one fatality, reported from rural forest areas of Chone and Flavio Alfaro counties, Manabí Province, western Ecuador.
Vasquez et al. (Vásquez et al., 2021)	2021	Case report	1	Pediatric patient with no prior medical history diagnosed with scorpionism; treatment included life support and symptomatic management, as anti-venom is unavailable in Ecuador.
Román et al. (Román et al., 2018)	2022	Cohort	20	Most patients exhibited leukocytosis and low serum potassium levels, with a significant negative correlation between the two. Scorpions from Macuma, Taisha County, were genetically related to *Tityus obscurus* from the Brazilian Amazon.
Ochoa-Andrade et al. (Ochoa-Andrade et al., 2022)	2022	Cohort	134	Stings most commonly occurred on upper and lower extremities (92.5%). Severe intoxication was observed in 12.7% of cases. The annual morbidity presentation was higher in 2017, the year in which 52.9% of all cases occurred.
Borges & Román (Borges and Román, 2023)	2023	Case report	1	A 4-month-old Ecuadorian boy of Shuar origin was stung by a scorpion (*Tityus cisandinus*) in Morona Santiago, presenting with pulmonary edema and systemic inflammation.

The Amazon region exhibits the highest incidence of scorpion stings, with 81.98 cases per 100,000 inhabitants. Among the provinces, Morona Santiago is the most affected, accounting for 35.50% of all cases and an incidence rate of 284.14 per 100,000. In this province, the scorpion species identified are *Ananteris ashmolei, Tityus asthenes, Tityus bastosi, Tityus demangei, Tityus ythieri, Chactas yaupi, Teuthraustes dubius, Teuthraustes festae, Troglotayosicus vachoni* (Brito and Borges, 2015). While in Sucumbíos, the second province with the highest incidence of scorpion stings, the species found are *Tityus asthenes*, *Tityus gasci*, and *Chactas moretii* (Brito and Borges, 2015). Previous studies on the Amazon have documented this issue. Román et al. (2018) reported 20 scorpion sting cases, including one death, over 2 years at a general hospital, while Ochoa Andrade et al. (2022) documented 134 stings between 2016 and 2018, of which 12.7% were classified as severe envenomations (Ochoa-Andrade et al., 2022; Román et al., 2018). Seasonal trends indicate that stings predominantly occur in October, April, December, and March. The months when the humid or rainy season occurs. (Ochoa-Andrade et al., 2022). Underreporting is likely in areas like Taisha canton, where cultural perceptions among indigenous populations, such as the Shuar, view scorpion stings as inevitably fatal, thus discouraging medical attention (Román et al., 2018).

The provinces with the lowest incidence of stings are Azuay, a region where the species *Hadruroides udvardyi* and *Teuthraustes gervaisii* are found, and the province of Cotopaxi, where the species *Tityus forcípula*, *Teuthraustes atramentarius*, *Teuthraustes oculatus*, and *Teuthraustes whymperi* are found. (Brito and Borges, 2015). Interestingly, no scorpion sting cases were reported from the Galapagos Islands during the study period. Two endemic scorpion species, *Centruroides exsul* and *Hadruroides galapagoensis*, are present in this region, but their stings are not clinically significant for humans (Borges, 2016). Additionally, the low population size (approximately 25,000 inhabitants spread across four of the 13 largest islands) and limited agricultural activities—known risk factors for scorpion stings—may contribute to the absence of reported cases (Guzman and Guzman, 2017; Trinidad-Porfirio et al., 2023).

Living in rural areas is the main risk factor for scorpion stings, as these areas often require storing firewood, leaves, tools, or construction materials near homes, which creates ideal habitats for scorpions. Additionally, collecting firewood increases the risk of stings due to prolonged exposure in the field and interaction with the ground. (Trinidad-Porfirio et al., 2023; Vasconez-Gonzalez et al., 2024). Regarding the climate, it has been observed that as the temperature increases, the incidence of stings also rises. Sting incidence is higher in the Amazon and Coastal regions with mean day temperature of 28C and 25C respectively compared to the Sierra (8C) (Ghorbani et al., 2021; Varela and Ron, 2022).

Our results revealed that women accounted for a higher proportion of sting cases (52%) compared to men (48%). These findings differ from study from Colombia, where Reyes-Vega reported 50.49% of cases in men, and Gómez et al. observed that 67% of cases occurred in men (Gómez et al., 2010; Reyes-Vega et al., 2021). Similarly, studies in Mexico reported higher incidences in men, Villegas-Arrizón et al., in their study where they surveyed 3294 people, found that the scorpion sting rate by sex was 20.7% for men and 8.7% for women, while Trinidad-Porfirio et al. reported 5.4% in men versus 3.5% in women (Trinidad-Porfirio et al., 2023; Villegas-Arrizón et al., 2009). This difference could be due to the percentage of men and women who seek medical services for health problems in Ecuador. According to data from the INEC, of 39.8 million consultations attended, 61.07% correspond to women and 38.93% to men (INEC, 2020).

In Ecuador, despite the existence of a clinical management guide for scorpion stings that includes the use of anti-scorpion serum, access to antivenom is severely limited. Antivenom is not included in the country's basic medication list (Borges et al., 2015; MSP, 2017; Ochoa-Andrade et al., 2022; Román et al., 2018; Vásquez et al., 2021). Studies have shown that administering antivenom within the first hours after a sting is highly effective, resolving clinical symptoms within 4 h and reducing circulating venom levels (Boyer et al., 2009). However, in Ecuador, management of scorpion stings is typically symptomatic, relying on benzodiazepine, analgesics, antiemetics, corticosteroids, and local anesthetics (Ochoa-Andrade et al., 2022; Vásquez et al., 2021). Emphasis on producing effective antivenoms for local species is urgently needed to improve outcomes.

## 5 Limitations

This study has several limitations. One of the primary constraints is the lack of information regarding the scorpion species responsible for the stings, preventing the identification of species most frequently involved in sting accidents. Additionally, the absence of data on symptoms and complications limits the ability to determine high-risk groups and identify scorpion species whose stings are more likely to cause severe outcomes.

Another important limitation is the potential underreporting of cases, particularly in rural areas where access to medical services is restricted. Furthermore, the recorded cases only include those deemed severe enough to warrant a visit to hospitals or health centers. Consequently, incidents where victims experienced mild symptoms and did not seek medical attention are not captured in the databases analyzed. This limitation may also explain the apparent overrepresentation of pediatric cases in the study.

## 6 Conclusion

Scorpionism remains a significant yet understudied public health concern in Ecuador. This study presents the first comprehensive nationwide epidemiological analysis, identifying the Amazon region as the most affected area, with Morona Santiago reporting the highest incidence rates.

Despite the existence of clinical management guidelines for scorpion stings, the limited availability of antivenom and its exclusion from the national essential medicines list poses significant challenges to effective treatment. Consequently, clinical management predominantly relies on symptomatic approaches, highlighting the urgent need for local anti-venom production and improved accessibility to address this pressing public health challenge.

Additionally, the underreporting of cases remains a 245 concern, particularly due to the lack of detailed epidemiological data. The existing bulletins do not include critical information such as the anatomical site of the sting, the scorpion species involved, or whether incidents occurred in urban or rural settings. To enhance data collection, the implementation of mobile technologies and the establishment of local reporting partnerships are recommended. Furthermore, continuous monitoring of scorpion species distribution is essential, as updated information is lacking. Such surveillance would help determine whether certain species have expanded into previously non-endemic areas.

This study underscores the necessity of strengthening epidemiological surveillance, increasing public awareness, and implementing policy reforms to mitigate the burden of scorpionism in Ecuador. Further research is needed to investigate regional disparities, improve clinical outcomes, and inform evidence-based public health interventions.

## CRediT authorship contribution statement

**Jorge Vasconez-Gonzalez:** Writing – original draft, Validation, Resources, Methodology, Investigation, Formal analysis, Data curation, Conceptualization. **Juan S. Izquierdo-Condoy:** Writing – review & editing, Visualization, Validation, Supervision, Project administration, Methodology, Investigation, Data curation. **Camila Miño:** Writing – original draft, Resources, Methodology, Investigation, Data curation. **María de Lourdes Noboa-Lasso:** Writing – original draft, Validation, Resources, Methodology, Investigation, Formal analysis, Data curation. **Esteban Ortiz-Prado:** Writing – review & editing, Validation, Supervision, Project administration, Methodology, Investigation.

## Funding

None to declare.

## Declaration of competing interest

The authors declare that they have no known competing financial interests or personal relationships that could have appeared to influence the work reported in this paper.

## Data Availability

No data was used for the research described in the article.
