# Corrigendum to “Epidemiological and geodemographic patterns of scorpionism in Ecuador: A nationwide analysis (2021–2024)” [Toxicon X 26(2025) 1–6/100218]

**DOI:** 10.1016/j.toxcx.2025.100241

**Published:** 2026-01-06

**Authors:** Jorge Vasconez-Gonzalez, Juan S. Izquierdo-Condoy, Camila Miño, María de Lourdes Noboa-Lasso, Esteban Ortiz-Prado

**Affiliations:** a One Health Research Group, Faculty of Health Science, Universidad de Las Americas, Quito, Ecuador; b Program in Occupational Safety and Health, The University of Porto, Porto, Portugal

The authors regret < Following publication, an error was identified in Figure 3. The map depicting the provincial distribution of scorpionism cases inadvertently shows an outdated version rather than the final approved figure. This error is limited solely to the graphical representation; all underlying data, analyses, interpretations, and conclusions remain entirely accurate and unaltered.

**Figure:**
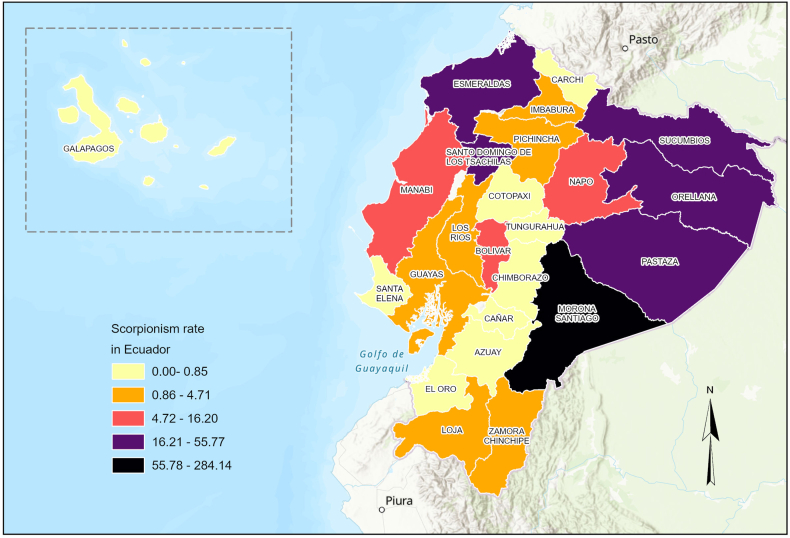

Fig. 3. Scorpion sting incidence rates per 100,000 inhabitants by province in Ecuador (2021–2024).>.

The authors would like to apologise for any inconvenience caused.

